# Proving Bone Marrow Plasma Cell Clonality by Flow Cytometry: An Important Tool in the Diagnosis of Immunoglobulin Light-Chain Amyloidosis

**DOI:** 10.3390/biomedicines13122925

**Published:** 2025-11-28

**Authors:** Sorina Nicoleta Badelita, Horia Mihail Sandu, Daniel Coriu, Andreea Jercan, Sinziana Barbu, Larisa Zidaru, Mihai Emanuel Himcinschi, Camelia Dobrea, Monica Popescu, Delia Codruta Popa

**Affiliations:** 1 Department of Hematology, Fundeni Clinical Institute, 022328 Bucharest, Romania; sorinabadelita@gmail.com (S.N.B.); popadeliacodruta@gmail.com (D.C.P.); 2 Department of Hematology, “Carol Davila” University of Medicine and Pharmacy, 050474 Bucharest, Romania; 3 Department of Biochemistry and Pharmacology, “Victor Babes” University of Medicine and Pharmacy, 300041 Timișoara, Romania; 4 OncoTeam Diagnostic, 012244 Bucharest, Romania; 5 Department of Pediatrics, “Carol Davila” University of Medicine and Pharmacy, 050474 Bucharest, Romania

**Keywords:** primary, amyloidosis, diagnostic tests, routine, methods, MRD, plasma cell dyscrasias, gammopathies, Congo Red, prognosis, retrospective studies, humans

## Abstract

**Background/Objectives**: Light-chain amyloidosis (AL Amyloidosis) is a rare systemic plasma cell disorder in which misfolded light chains form amyloid fibrils that damage vital organs. Diagnosis is often delayed because of variable presentation and overlap with other plasma cell diseases. **Methods**: We retrospectively analyzed 23 patients diagnosed between 2022 and 2025 at the Fundeni Clinical Institute. All cases underwent free light-chain testing, serum immunofixation, immunohistochemistry, and bone marrow analysis. Since 2022, next-generation flow cytometry (NGF) has been applied to detect clonal plasma cells and characterize their immunophenotype. **Results**: NGF confirmed clonality in 21 of 23 patients (91.3%), including two cases undetected by conventional assays. Serum immunofixation was positive in 69.6% and immunohistochemistry in 78.3%. Among clonal cases, λ restriction predominated (71.4%). Detection was highest when NGF was combined with standard methods, especially in patients with dFLC < 100, where negative immunofixation was more frequent (*p* = 0.027). **Conclusions**: Multiparameter flow cytometry (MFC) provides highly sensitive detection of small clonal plasma cell populations often missed by standard assays, offering a valuable tool for early and accurate diagnosis of low-burden plasma cells in AL amyloidosis. Integrating MFC into routine evaluation improves diagnostic accuracy, identifies overlooked clonal populations, and supports earlier, more informed clinical decisions.

## 1. Introduction

Light-chain (AL) amyloidosis is a very rare (orphan status in both the EU and US [1]) systemic plasma cell disorder in which circulating light chains misfold and assemble into amyloid fibrils. These fibrils deposit in vital organs, such as the heart, kidneys, and liver, leading to progressive and often irreversible dysfunction. Unlike the localized form, which remains confined to a single site [2], systemic AL amyloidosis affects multiple organs and carries a poor prognosis if not diagnosed early.

Early diagnosis is not the norm; on the contrary, most patients will visit more than four physicians before obtaining the right diagnosis and starting the specific treatment [3], but it is vital in order to prevent irreversible organ damage [4]. Underdiagnosis is unfortunately frequent and could be found in up to 30% of patients presenting with irreversible organ damage [5]. The median time from symptom onset to diagnosis was found to be 2.7 years in a big US retrospective study and approximately 7 months (range 0–61 months) in a single-center cohort from Boston [6,7]. The clinical picture is heterogeneous and overlaps with several conditions, including presentation as extremely rare forms [8]. Secondary (AA) amyloidosis, hereditary transthyretin amyloidosis, and the wild-type form can closely mimic AL amyloidosis, making accurate amyloid typing and imaging techniques essential [9]. Further complexity arises from its resemblance to other plasma cell dyscrasias, including monoclonal gammopathy of undetermined significance (MGUS), smoldering myeloma (SM), multiple myeloma (MM), and plasma cell leukemia (PCL), and the lower infiltration of bone marrow by clonal plasma cells (<10%) in the bone marrow when compared to multiple myeloma or AL amyloidosis coexistent with multiple myeloma, which can further be accompanied by negative serum electrophoresis in up to 30% of patients [10,11].

Distinguishing AL amyloidosis from these disorders requires strict diagnostic criteria established by the International Myeloma Working Group (IMWG): (i) presence of an amyloid-related syndrome, (ii) histopathological confirmation of amyloid deposits by Congo Red Staining, (iii) amyloid typing to ensure it has light-chain origin, and (iv) evidence of plasma cell clonality [12]. Note: The λ:κ case ratio should not be confused with the κ/λ ratio. The λ:κ case ratio represents the number of patients with λ or κ clonality, whereas the κ/λ ratio refers to the quantitative relationship between free light-chain concentrations in serum.

Amyloid fibrils can have different proteins as progenitors of disease; all of them will stain positive when reacted with Congo Red staining in a nonspecific way. In order to find what type of protein determined the amyloid deposits, different types of tests must be used, either antibody-dependent or antibody-independent techniques. Immunohistochemistry, immunoelectron microscopy, and immunofluorescence are part of the first category, while genetic and proteomic assays belong to the second category [13,14]. Mass spectrometry is currently considered the gold standard for amyloid typing, but its limited availability restricts widespread use. Thus, a combined approach using conventional methods with complementary techniques remains essential in daily practice [13,15].

Multiple laboratory methods are available to demonstrate clonality, yet none is fully reliable on its own. Serum-free light-chain assays, immunofixation, and immunohistochemistry remain standard, but all may yield inconclusive results.

Despite comprehensive overviews of AL amyloidosis diagnosis in the recent literature, flow cytometry remains underrepresented. For instance, a 2024 New England Journal of Medicine review detailing current diagnostic pathways omits multiparameter flow cytometry entirely, underscoring the gap between established myeloma workflows and their adoption in AL amyloidosis [16].

It is important to emphasize the fact that MFC does not directly identify amyloid fibrils, but rather supports the AL Amyloidosis diagnosis by demonstrating a clonal plasma cell population in the bone marrow, thereby indirectly inferring a light-chain origin of the amyloid deposits.

Multiparameter Flow Cytometry (MFC) has gained increasing relevance as a sensitive tool for detecting minimal clonal plasma cell populations [17]. Although MFC is well established for diagnosing and monitoring plasma cell dyscrasias, such as multiple myeloma, this study focuses on its specific utility in the diagnostic evaluation of light-chain (AL) amyloidosis. We aim to demonstrate how integrating MFC into the diagnostic algorithm enhances accuracy in routine clinical practice, particularly in low plasma cell burden cases where clonality is difficult to detect by conventional methods.

## 2. Materials and Methods

**Scheme 1:**
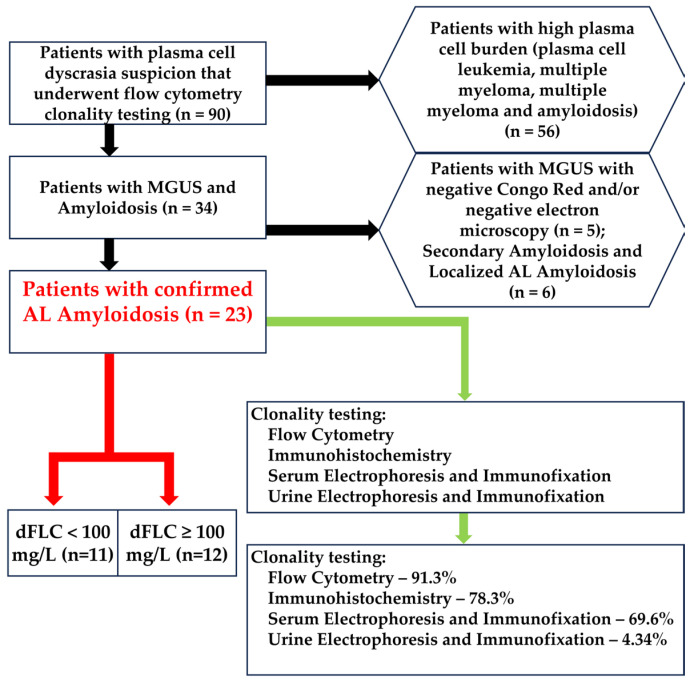
Selection methods for our cohort. In the hexagons are represented the exclusion criteria. The red arrows represent the dFLC grouping. The green arrow represents the clonality tests. From the total of 90 patients with plasma cell dyscrasia suspicion that also underwent flow cytometric testing, 56 were excluded because of the very high plasma cell burden, 5 were excluded because the underlying pathology was demonstrated to be MGUS with negative amyloid deposition by Congo Red staining and/or electron microscopy, and another 6 had either localized or secondary amyloidosis.

This retrospective study includes patients with light-chain (AL) amyloidosis diagnosed between 2022 and 2025 in the Hematology Department of the Fundeni Clinical Institute. A schematic overview of how patients were selected is represented in Scheme 1.

Five sample types were collected: (i) peripheral blood in SST tubes (BD Biosciences, San Jose, CA, USA) for creatinine measurement, free light-chain assay, and serum electrophoresis; (ii) first and second pulls of bone marrow aspirate in K2-EDTA tubes (BD Biosciences, San Jose, CA, USA) for flow cytometry analysis and bone marrow smears; (iii) bone marrow biopsy for immunohistochemistry; (iv) 24 h urine in sterile containers for urine electrophoresis and immunofixation; (v) fine needle aspiration of fat tissue for Congo Red staining.

**Figure 1:**
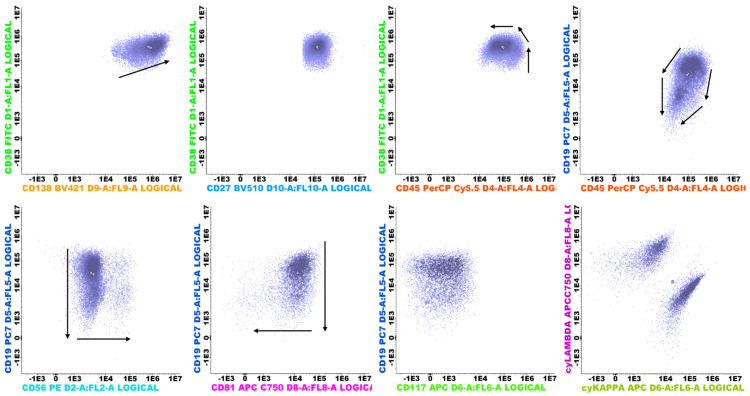
Normal polyclonal plasma cells (purple) and their maturation pathways, represented as black arrows.

**Figure 2:**
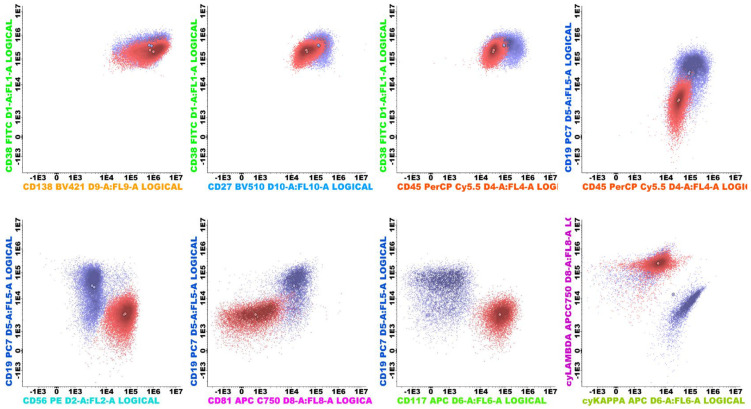
Clonal plasma cells with Lambda light-chain restriction (red) with aberrant immunophenotype (CD56++, CD117++, CD81 −in comparison to the normal plasma cells (purple). The normal plasma cells can be present even when a clonal population is concomitantly present (both Figure 1 and Figure 2 are from the same sample) and act as an internal control for the analysis.

Flow cytometric analysis was performed on all patients. In order to minimize the costs and implementation time, we opted to use the already established panel for multiple myeloma that we use in our laboratory. The first bone marrow aspirate was assessed in all cases. Between 1 and 2 mL of bone marrow was collected into a single 5 mL K2-EDTA tube. Samples were processed within 24 h of aspiration in all cases. Pre-analytical procedures followed the EuroFlow protocol: the Multiple Myeloma Minimal Residual Disease(MM MRD KIT) (Cytognos S.L., Salamanca, Spain; BD Biosciences, San Jose, CA, USA, REF: CYT-MM-MRD8) was used to stain all samples (CD38 FITC multiepitope, CD56 PE clone C5.9, CD45 PerCP-Cyanine5.5 clone EO1, CD19 PE-Cyanine7 clone SA287, CD117 APC clone 104D2, CD81 clone APC-C750, CD138 BV421 clone MI15, CD27 BV510 clone O323, cyIgKappa APC and cyIgLambda APC-C750). The number of events interrogated exceeded 5 × 10^6^ per sample. A bulk-lysis protocol was applied to maximize plasma cell recovery. Data were acquired on two 13-color instruments: BD FACSLyric (BD Biosciences, San Jose, CA, USA) and DxFLEX (Beckman Coulter, Brea, CA, USA). FCS 3.0 files were analyzed individually using Infinicyt software version 2.0.6 (Cytognos S.L., Salamanca, Spain). Automated analysis using the Infinicyt MM MRD database was not employed; files were reviewed manually. The primary endpoints were (i) clonality demonstrated by cytoplasmic κ or λ Light-chain restriction and (ii) pathology-related immunophenotypic abnormalities (CD56 overexpression; underexpression of CD27, CD81, and CD45; CD117 positivity; and under-/overexpression of CD138 and CD38) in plasma cells (Figure 1 and Figure 2) [18]. Dead cells and debris were excluded from analysis by employing different analytical methods: Doublet exclusion by both Side Scatter Area versus Side Scatter Height and Forward Scatter Area versus Forward Scatter Height, and Debris (small events with low internal complexity) from Side Scatter Area versus Forward Scatter Area.

Serum protein electrophoresis (sPEP), urine protein electrophoresis (uPEP), serum immunofixation (sIFE), and urine immunofixation (uIFE) were performed on agarose gels to separate and quantify serum fractions, with attention to monoclonal bands, followed by immunofixation (Hydragel 4, Sebia, Évry-Courcouronnes, France) to identify heavy- and light-chain components. Urine analysis was performed on the 24 h collected urine to ensure good sensitivity for low-level monoclonal protein detection. Serum-free light-chain (sFLC) analysis was performed using κ and λ reagents on the Optilite turbidimetric system (The Binding Site, Birmingham, UK) and the BN ProSpec nephelometer (Siemens Healthineers, Erlangen, Germany), yielding κ, λ, and κ/λ ratio values. Together, these assays enabled the detection and characterization of monoclonal gammopathies.

Bone marrow biopsy (BMB) sections (2.5 µm) were stained with Hematoxylin Eosin and Congo Red (Congo Red staining kit, with Ventana Benchmark special stain automated, Roche Diagnostics GmbH, Mannheim, Germany). Automated immunohistochemistry (Ventana Benchmark Ultra, Roche Diagnostic) was performed, with antibodies against CD138 (clone B-A38 RTU, Cell Marque), κ light chain (Rabbit polyclonal primary antibody, RTU, Roche Diagnostic), and λ light chain (Rabbit Polyclonal primary antibody, RTU, Roche Diagnostic). Detection used an HRP polymerase system with DAB chromogen, enabling the assessment of a plasma cell infiltrate (CD138) and light-chain expression and restriction in plasma cells.

Abdominal fat pad biopsy sample slides were examined under polarized light for characteristic apple green birefringence. Each staining batch included a known amyloid-positive tissue section as a positive control and an internal negative control consisting of normal adipose tissue.

Statistical analyses were performed using SPSS v26 (IBM Corp., Armonk, NY, USA) and Microsoft Excel (Microsoft Corp., Redmond, WA, USA). Normality was assessed by using the Shapiro–Wilk test. Descriptive statistics are reported as mean ± SD, median (interquartile range, IQR) for continuous variables, and *n* (%) for categorical variables. Correlations between normally distributed continuous variables were assessed with Pearson’s correlation; for non-normal distributions, Spearman’s rank correlation was used. Differences between groups were tested using the independent samples *t*-test for normally distributed continuous variables and the Mann–Whitney U test for non-normal continuous variables. Fisher’s exact test and the χ^2^ test were used for categorical variables. Statistical significance was set at two-tailed *p* < 0.05.

## 3. Results

Between 2022 and 2025, 23 patients were diagnosed with AL amyloidosis in the Hematology Department of the Fundeni Clinical Institute. The cohort included 23 patients, of whom 12 were female (52%) and 11 were male (48%), reflecting a balanced sex distribution. Approximately 70% originated from urban areas, while 30% were from rural regions. The mean age at disease onset was 66 years, and the mean interval between symptom onset and confirmed diagnosis was 9 months, underscoring the diagnostic delay commonly encountered in AL amyloidosis.

Regarding organ involvement, renal impairment was present in 70% of patients, cardiac involvement in 43%, and neuropathy (including both somatic and autonomic forms) in 57%. Other organ manifestations, such as cutaneous, digestive, hepatic, pulmonary, or soft tissue amyloidosis, were documented in 43% of cases. These organ dysfunctions frequently co-occurred, as multiple systems were often affected simultaneously in the same patient.

The cohort’s characteristics are presented in Table 1. For an easier visual interpretation, the corresponding figures are in the Supplementary Material (Figures S1–S8).

### 3.1. Cohort Clonality and Diagnostic Characteristics (n = 23)

**Figure 3:**
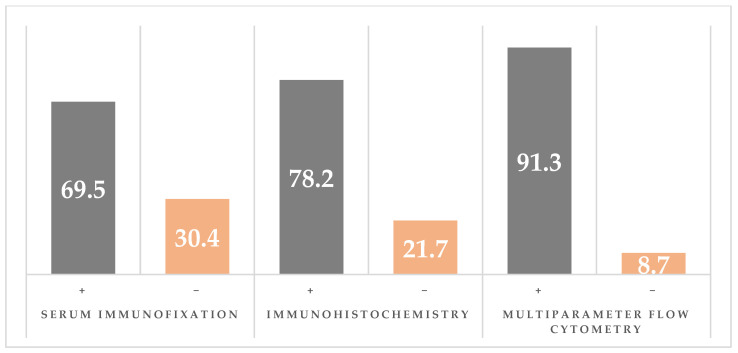
Comparison of clonality detection by serum immunoelectrophoresis (sIFE), immunohistochemistry (IHC), and multiparameter flow cytometry (MFC).

Congo Red staining of abdominal fat or salivary glands was positive in 22/23 (95.7%) (Figure S9). Serum immunofixation was positive in 16/23 patients (69.6%) for either κ or λ. Urine immunofixation was positive in only 1/23 patients (4.34%). Clonality by immunohistochemistry has demonstrated clonal plasma cells in 18/23 (78.3%). Flow cytometry clonality assessment identified κ and/or λ restriction in 21/23 (91.3%) patients; in 2/23 (8.7%), plasma cells were normal/reactive. Among the 21 clonal cases, λ restriction occurred in 15/21 (71.4%), κ restriction in 5/21 (23.8%), and dual κ + λ clones in 1/21 (4.8%). The λ:κ case ratio was 3:1 (Figure S10). The comparison between methods for clonality identification is presented in Figure 3.

Even though flow cytometry identified clonality in 91.3% cases, immunohistochemistry in 78.3% cases, and serum immunofixation in 69.6%, when all three tests were combined, clonality was demonstrated in all cases.

### 3.2. Grouping of by dFLC < 100 mg/L (n = 11) and dFLC ≥ 100 mg/L (n = 12)

To further characterize the cohort, we descriptively compared patients with lower versus higher dFLC at diagnosis. In Table 2, the clonality by different methods is investigated.

A pragmatic cut-off of dFLC = 100 mg/L defined two groups: dFLC < 100 mg/L (*n* = 11) (Table 3) and dFLC ≥ 100 mg/L (*n* = 12) (Table 4). The corresponding figures can be visualized in the Supplementary Figures (Figures S11–S16).

Associations between dFLC groups and clonality detection were assessed using Fisher’s exact test; the small cohort limits statistical power but confirms a significant trend (*p* = 0.027).

**Figure 4:**
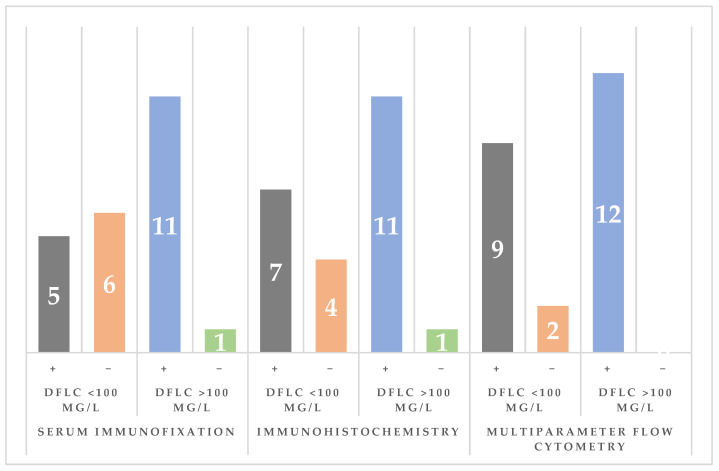
Comparison of clonality detection by serum immunoelectrophoresis (sIFE), immunohistochemistry (IHC), and multiparameter flow cytometry (MFC) across dFLC groups (using the 100 mg/L cutoff value; in gray and orange are represented the number of cases in the dFLC < 100 mg/L group while blue and green are used to represent the number of cases in the dFLC > 100 mg/L group).

Patients with dFLC < 100 mg/L were more likely to have negative immunofixation (55% vs. 8%), whereas those with dFLC ≥ 100 mg/L were more likely to have evidence of clonality by serum immunofixation (92% vs. 45%). The odds of negative immunofixation were higher in the dFLC < 100 mg/L group (OR = 13.2; 95% CI, 1.24–140.7). Immunohistochemistry demonstrated clonality in 7/11 patients with dFLC < 100 mg/L (63.6%) and 11/12 with dFLC ≥ 100 mg/L (91.7%). Flow cytometry identified clonal plasma cells in 9/11 (81.8%) and 12/12 (100%) patients in the dFLC < 100 mg/L and dFLC ≥ 100 mg/L groups, respectively (Figure 4).

Congo Red of abdominal fat was positive in 10/11 (90.9%) vs. 12/12 (100%).

Another significant difference is that λ was significantly higher in the dFLC ≥ 100 mg/L group (*p* = 0.002) (Table 3 and Table 4). This difference appears naturally, given the commonality of λ chain involvement in AL amyloidosis compared to κ light chain (3:1 case ratio in favor of λ).

## 4. Discussion

Light-chain amyloidosis is a rare disorder with a broad spectrum of clinical features that overlap with many other diseases. Demonstrating clonality is a key criterion in differentiating AL amyloidosis from other amyloidosis diseases, underscoring the need for multiple complementary testing methods. Guidelines recommend that suspected AL amyloidosis be evaluated with sPEP, sFLC, serum, and urine immunofixation (sIFE and uIFE), including free κ and λ assessment [19,20].

The median time from symptom onset to diagnosis in our cohort was 7 months, which aligns with or slightly improves upon previously reported delays. In a large U.S. retrospective study, the median diagnostic interval was 2.7 years, while a single-center Boston series reported a median of 7 months (range 0–61 months) [6,7]. These comparisons illustrate that, although diagnostic awareness has improved, a delay of nearly a year remains common and continues to contribute to organ impairment at presentation.

When the involved light chain is produced in low quantities, serum and urine tests may yield inconclusive results (for serum and urine immunoglobulin quantification, we use an indirect method using electrophoresis, immunofixation, and total protein values; for urine analysis, urine immunofixation was performed on 24 h collected and concentrated urine; urinary free light chain could also be tested, but it is not a routine analysis in our facility, so no data was extracted for this cohort); urine immunofixation is frequently negative or very low positive, especially in patients without concomitantly renal impairment, which is also the case in our cohort, where only one patient had a positive urine immunofixation [13]. Uncertain findings can be further amplified or appear standalone when the patient suffers simultaneously from a renal dysfunction (which will increase both polyclonal and involved light chain in the serum) or from an inflammatory syndrome (which will increase the polyclonal light chain in the serum) [21,22,23]. When the serum or urine electrophoresis and immunofixation are inconclusive, clonality must be further investigated by immunohistochemistry and flow cytometry. In this setting, flow cytometry has a set of advantages: independence from M-protein quantity/type and renal function, and sensitivity down to 10^−5^–10^−6^ [17,24], complementing immunohistochemistry and immunofixation by detecting residual or low-frequency clonal plasma cells that may be missed by conventional assays.

In our cohort, the flow cytometry yielded the highest clonality detection rate (κ and/or λ) at 91.3%, compared with SIFE (69.6%) and IHC (78.3). It also detected clonality in two patients who were negative by all other methods (both patients had dFLC < 100 mg/L). Bone marrow smears showed >5% plasma cells in both, but most cells were normal/reactive. These findings underscore the ability of flow cytometry to distinguish clonal plasma cells from coexisting normal plasma cells. On the other hand, flow cytometry missed two patients; one patient had clonality proven by both IHC and SIFE, while the other patient was only IHC-positive, underscoring the complementary effect of introducing flow cytometry to routine diagnostic work-up and not the straight-up replacement of other methods. This effect could be attributed to the fact that sometimes bone marrow aspirate does not contain plasma cells due to their anatomic placement in the plasma cell niche or due to the loss of plasma cells during preanalytical preparation [25]. Immunohistochemistry also comes in handy in the differential diagnosis of AL amyloidosis. For example, Transthyretin (TTR) and Serum Amyloid A (SAA) can be identified on both bone marrow biopsies and organ biopsies, usually invalidating an AL amyloidosis diagnosis and increasing suspicion for the respective types of amyloidosis (in our cohort, all patients had negative Anti-TTR and Anti-SAA).

As mentioned before, flow cytometry was able to identify clonal plasma cells in two patients who presented with very similar features. Both patients had single renal impairment due to amyloid deposits, identified by renal biopsy with positive Congo Red staining. In one case, immunofluorescence was positive and revealed λ light-chain restriction consistent with the flow cytometry findings. In the other, the immunofluorescence was negative despite κ light-chain restriction detected by flow cytometry. Immunohistochemistry of the bone marrow for light-chain clonality was negative in both cases, and the immunofixation of serum and urine was unable to prove the clonality. The time from renal biopsy to diagnosis also differed: 8 months for the λ-positive patient and 3 months for the κ-positive patient. This difference further suggests the importance of incorporating flow cytometry when initial testing results are inconclusive, as it may facilitate earlier diagnosis and timely initiation of therapy. Congo Red staining identifies amyloid fibrils but is not able to distinguish their underlying cause, making complementary methods, such as MFC, essential for determining the source of Congo Red positivity. The technique is also very slow, usually requiring more than three weeks for a final result, whereas MFC can provide clonality proof in under 24 h. Because the amyloidosis deposit confirmation is only the first step, rapidly defining the pathogenic mechanism is crucial: secondary amyloidosis requires suppression of the inflammatory trigger, while AL amyloidosis demands the elimination of the clonal plasma cell population to halt ongoing fibril production.

Fine needle aspiration can be a practical option for evaluating localized AL amyloidosis when tissue access is limited. If paired with flow-cytometric light-chain analysis, it could allow for a rapid confirmation of plasma cell clonality and reduce the need for more invasive procedures. This combined approach may complement immunohistochemistry and improve the diagnostic workflow for patients with small or superficial lesions [26].

Recent findings in tumor immunology indicate that specific circulating immune cells may influence the appearance or persistence of abnormal plasma cell populations. Studies using Mendelian randomization in osteosarcoma showed that distinct immune cell subsets can modify disease risk [27]. Exploring whether a similar relationship exists in AL amyloidosis could help identify early immune signatures that signal a higher probability of developing clonal plasma cell activity or organ damage.

To better understand where our results are positioned in the context of recent scientific findings, a selection of papers was gathered and compared. In the following paragraphs, the results used for comparison will be listed first, and the values that characterize our series of patients are in parentheses. Lambda Light chain has been found more often than Kappa Light chain to be the driver of amyloid fibril deposition because of its structural instability [16]. These patients have a higher risk of organ dysfunction, especially cardiac and renal dysfunctions.

In a large cohort of 98 patients, the λ:κ case ratio was 1.88:1, a value lower than the 3:1 observed in our series. Unlike the λ:κ case ratio, other assays’ results are closer: immunofixation was positive in 63.3% cases (vs. 69.6%), immunohistochemistry identified clonality in 85.7% cases (vs. 78.3%), and flow cytometry identified clonal plasma cells in 92.9% cases (vs. 91.3%) [28].

In another large 94-patient series, serum immunofixation was positive in 93% cases (vs. 69.6%), and flow cytometry identified clonal plasma cells in 99% cases (vs. 91.3%). Differences may reflect referral center bias and database-driven analysis. Other interesting points of comparison that are worth noting are mean dFLC = 171 mg/L (vs. 172 mg/L), and mean bone marrow plasma cell percentage was 8% (vs. 8.2%), suggesting a close resemblance between the population’s characteristics [29].

The most similar results appear when compared to the results obtained by Lisenko et al. [30]. In this medium-sized cohort composed of 63 patients, the λ:κ cases ratio is 2.93:1, very similar to our 3:1 case ratio. Serum Immunofixation was positive in 78% patients (vs. 69.57%), immunohistochemistry of bone marrow was able to detect clonal plasma cells in 89% of the cases (vs. 78.26%), and the multiparameter flow cytometer was able to identify a clonal plasma cell in 97% cases (vs. 91.3%).

An immunohistochemistry-oriented study reported on their cohort of 76 patients a λ:κ distribution of 1:1.6 (κ predominance must be noted in this case), with 68% unequivocally κ or λ positive. These results differ drastically from all the results found and could be interpreted as an outlier [31].

Long non-coding RNAs have an increasingly recognized role in controlling gene expression in malignant plasma cells. Investigating the presence of disease-specific lncRNAs in AL amyloidosis could provide new information about how amyloidogenic clones emerge. Previous work on lncRNA Gm2044 illustrates how this type of molecule can participate in pathological processes and may serve as a potential biomarker in future studies [32].

On a small cohort of 35 patients that closely replicates our conditions, the research group demonstrated that baseline multiparameter flow cytometry had a prognostic value for overall survival (OS) using a ≤1% vs. >1% bone marrow plasma cell cutoff (2-year OS, 90% vs. 44%). Patients with >5% vs. ≤5% normal plasma cells within total BMPC also showed differential 2-year OS (88% vs. 37%; *p* = 0.01). These data indicate that, beyond clonality, multiparameter flow cytometry (MFC) informs prognostic stratification. In this paper, flow cytometry identified clonal plasma cells in 97% cases [24].

The prognostic value that flow cytometry brings was also further evaluated and confirmed by Muchtar et al. [33]. Their research group reported that clonal plasma cells >2.5% by MFC were associated with shorter progression-free survival (PFS) and OS; the 0.1% cut-off value for the residual clonal plasma cells at the end of the first-line therapy proved to be an efficient separator between the patients with inferior PFS and OS (>0.1%) and the patients with higher PFS and OS (≤0.1%).

Parallel to advancements in immunophenotyping by flow cytometry, new scientific boundaries were created by the implementation of proteomic analysis in amyloid typing. Immunoelectron microscopy (IEM) and mass spectrometry (MS) are two techniques that provide highly accurate results. To support this statement, a study on 106 Congo Red-positive biopsies was performed, and IEM identified amyloid deposition in 91.6%, while MS identified an amyloid signature in 98.1%; when combined, the techniques achieved 100% typing, illustrating their complementarity. Unfortunately, these techniques are still very expensive and require highly specialized and trained personnel, making the combination of immunofixation, immunohistochemistry, and flow cytometry a reliable, practical alternative [13].

Multiple studies examined associations between post-treatment bone marrow minimal residual disease (MRD) and organ responses, particularly cardiac and renal responses, as well as progression-free survival (PFS) and overall survival (OS). Although MRD is not yet an established biomarker in primary amyloidosis, its value has increased in treatment monitoring, given the fact that even very small quantities of clonal plasma cells in the bone marrow can secrete very small amounts of amyloidogenic light chain that exert a toxic effect on different organs and systems [34]. The toxicity of amyloid fibrils is well recognized and involves multiple mechanisms of tissue injury. One interesting pathway is the amyloid’s ability to trigger neutrophils’ release of neutrophil extracellular traps (NETs), which in turn generate reactive oxygen species (ROS) and amplify local inflammation at sites of amyloid deposition [35,36].

Reference centers began the use of next-generation flow cytometry to better predict organ dysfunction by MRD testing [37]. Naturally, our center will develop on the same pathway, and further studies on MRD in AL amyloidosis will search for a stronger link between MRD negativity and improved organ response. For MRD testing, the evidence is heterogeneous. Some articles found no correlation between MRD status and cardiac response, kidney response, PFS, or OS [38,39]. Other research groups found that MRD negativity is correlated to a better outcome on the cardiac response and PFS, while renal response and OS remained unaffected [40,41]. Muchtar et al. found that MRD negativity status is correlated with a better renal response and PFS [42]. Paladini et al. demonstrated that MRD negativity is correlated to both increased cardiac and renal response, and also to higher PFS [43].

## 5. Conclusions

Patients with AL amyloidosis and low dFLC (<100 mg/L) at diagnosis often have negative serum immunofixation (*p* = 0.027). This may delay timely diagnosis. Flow cytometry is a valuable tool for diagnosis. It enables the detection of small clonal plasma cell populations. The turnaround time is short (results within <6 h). Detection rates exceeded 90% in the present cohort and in several published series, making it a reliable testing method, but must be used in complementarity to other clonality tests like immunohistochemistry and serum and urine immunofixation in order to find proof of clonality in 100% cases (in centers lacking mass spectrometry, pairing flow cytometry with IHC and immunofixation of urine and serum can provide sufficient diagnostic certainty, minimizing the need for additional complex assays). This study is limited by its retrospective design and the relatively small cohort size (*n* = 23). Larger prospective, multicenter studies are required to validate these results and to establish standardized flow cytometry criteria for routine implementation. In particular, studies investigating the relationship between organ response and minimal residual disease will be essential to determine the role of flow cytometry as a validated biomarker in AL amyloidosis.

## Figures and Tables

**Table 1:** Cohort’s tested parameters (*n* = 23). In order, the assessed parameters are bone marrow plasma cell percentage by bone marrow smear (BMPC), dFLC (mg/L), κ and λ light chain (mg/L), κ/λ ratio, plasma cell percentage by flow cytometry (FCPC), serum creatinine (mg/dL) (sCREA), and eGFR (mL/min/1.73 m^2^).

	BMPC	dFLC	κ	λ	κ/λ Ratio	FCPC	sCREA	eGFR
Mean	8.57	172	60	164	1.98	1.17	1.26	66
SD	4.18	219	96	215	4.2	1.62	0.7	24
Median	10	125	21	88	0.26	0.6	1.05	69
Interquartile range	5–12	11–202	13–62	26–229	0.09–1.69	0.1–1.2	0.86–1.31	52–85
Min	0	0	6	14	0.01	0.03	0.68	15
Max	17	941	443	948	16	6	3.65	112
Range	17	941	436	933	16	5.98	2.97	97

**Table 2:** Clonality multi-crosstabulation table. Clonality was assessed by multiparameter flow cytometry (MFC), immunohistochemistry (IHC), and serum immunofixation (sIFE). No patients were found with all negative tests.

						dFLC < 100 mg/L	dFLC ≥ 100 mg/L
						Count	Count
Clonality by MFC	−	Clonality by IHC	−	Clonality by sIFE	−	0	0
					+	0	0
			+		−	1	0
					+	1	0
	+		−		−	2	0
					+	2	1
			+		−	3	1
					+	2	10

**Table 3:** dFLC < 100 mg/L group (*n* = 11). In order, the assessed parameters are bone marrow plasma cell percentage by bone marrow smear (BMPC), κ and λ light chain (mg/L), κ/λ ratio, plasma cell percentage by flow cytometry (FCPC), serum creatinine (mg/dL) (sCREA), and eGFR (mL/min/1.73 m^2^).

	BMPC	κ	λ	κ/λ Ratio	FCPC	sCREA	eGFR
Mean	8.18	51	45	1.35	0.83	1.46	67
SD	3.25	40	28	0.80	1.15	0.96	31
Median	10	34	35	1.40	0.45	1.05	76
Interquartile range	5–11	20–95	21–75	0.96–1.71	0.12–1	0.83–2	30–88
Min	3	10	16	0.14	0.03	0.68	15
Max	12	125	93	3.04	4	3.65	112
Range	9	114	77	2.90	3.98	2.97	97

**Table 4:** dFLC ≥ 100 mg/LL group (*n* = 11). In order, the assessed parameters are bone marrow plasma cell percentage by bone marrow smear, κ and λ light chain (mg/L), κ/λ ratio, plasma cell percentage by flow cytometry, serum creatinine (mg/dL), and eGFR (mL/min/1.73 m^2^).

	BMPC	κ	λ	κ/λ Ratio	FCPC	sCREA	eGFR
Mean	8.92	68	272	2.56	1.49	1.08	66
SD	5.017	129	255	5.83	1.96	0.24	18
Median	9.00	15	195	0.1	0.85	1.04	66
Interquartile range	5–13	9–53	141–407	0.02–0.22	0.17–1.8	0.86–1.3	53.5–75.5
Min	0	6.38	14	0.01	0.05	0.84	34
Max	17	443	948	16.56	6	1.56	97
Range	17	436	933	16.55	5.95	0.72	63

## Data Availability

The data presented in this study are available upon request from the corresponding author. The data are not publicly available due to ethical and privacy restrictions.

## Supplementary Materials

The following supporting information can be downloaded at https://www.mdpi.com/article/10.3390/biomedicines13122925/s1: Figure S1: Plasma-cell percentage by bone marrow smear; Figure S2: Distribution of dFLC values; Figure S3: Serum κ light-chain concentration; Figure S4: Serum λ light-chain concentration; Figure S5: Serum κ/λ ratio; Figure S6: Plasma cell percentage in bone marrow by flow cytometry; Figure S7: Serum creatinine in AL amyloidosis patients; Figure S8: Estimated glomerular filtration rate (eGFR) in AL amyloidosis patients; Figure S9: Congo red staining of abdominal fat and salivary gland; Figure S10: Distribution of κ and λ light-chain clonality detected by flow cytometry; Figure S11: Plasma-cell percentage by bone marrow smear in dFLC subgroups; Figure S12: Plasma cell percentage by flow cytometry in dFLC subgroups; Figure S13: Serum κ light chain quantification in dFLC subgroups; Figure S14: Serum λ light chain quantification in dFLC subgroups; Figure S15: Serum κ/λ ratio in dFLC subgroups; Figure S16: Serum creatinine in dFLC subgroups.

## Abbreviations

The following abbreviations are used in this manuscript:

AL	Light-chain Amyloidosis
IHC	Immunohistochemistry
sPEP	Serum protein electrophoresis
sIFE	Serum immunofixation
uPEP	Urine protein electrophoresis
uIFE	Urine immunofixation
IEM	Immunoelectron microscopy
MS	Mass Spectrometry
PFS	Progression Free Survival
OS	Overall Survival
dFLC	Difference between involved and uninvolved Light Chain
MRD	Minimal Residual Disease
MFC	Multiparameter Flow Cytometry

## References

[1] Kumar N., Zhang N.J., Cherepanov D. (2022). Global Epidemiology of Amyloid Light-Chain Amyloidosis. Orphanet J. Rare Dis..

[2] Martínez J.C., Lichtman E.I. (2022). Localized Light Chain Amyloidosis: A Self-Limited Plasmacytic B-Cell Lymphoproliferative Disorder. Front. Oncol..

[3] Gertz M.A., Dispenzieri A. (2020). Systemic Amyloidosis Recognition, Prognosis, and Therapy: A Systematic Review. JAMA.

[4] Zidaru L., Jercan A., Barbu S. (2025). DRD vs. D-VCD in Newly Diagnosed AL Amyloidosis: A Retrospective Single-Center Study. Doc. Haematol. Rev. Romana Hematol..

[5] Palladini G., Merlini G. (2016). What Is New in Diagnosis and Management of Light Chain Amyloidosis?. Blood.

[6] Hester L.L., Gifkins D.M., Bellew K.M. (2021). Diagnostic Delay and Characterization of the Clinical Prodrome in AL Amyloidosis among 1523 US Adults Diagnosed between 2001 and 2019. Eur. J. Haematol..

[7] Schulman A., Connors L.H., Weinberg J. (2020). Patient Outcomes in Light Chain (AL) Amyloidosis: The Clock Is Ticking from Symptoms to Diagnosis. Eur. J. Haematol..

[8] Pompilian V.M., Tanaseanu S., Badea C. (2017). IgG, Kappa Monoclonal Gammopathy of Unknown Significance with AL Amyloidosis Simulating Giant Cell Arteritis. Rom. J. Intern. Med..

[9] Gherghe M., Lazar A.M., Sterea M.C. (2023). Quantitative SPECT/CT Parameters in the Assessment of Transthyretin Cardiac Amyloidosis-A New Dimension of Molecular Imaging. J. Cardiovasc. Dev. Dis..

[10] Kourelis T.V., Kumar S.K., Gertz M.A. (2013). Coexistent Multiple Myeloma or Increased Bone Marrow Plasma Cells Define Equally High-Risk Populations in Patients with Immunoglobulin Light Chain Amyloidosis. J. Clin. Oncol..

[11] Rubinstein S.M., Stockerl-Goldstein K. (2021). How to Screen for Monoclonal Gammopathy in Patients with a Suspected Amyloidosis. JACC CardioOncol..

[12] Rajkumar S.V., Dimopoulos M.A., Palumbo A. (2014). International Myeloma Working Group Updated Criteria for the Diagnosis of Multiple Myeloma. Lancet Oncol..

[13] Abildgaard N., Rojek A.M., Møller H.E. (2020). Immunoelectron Microscopy and Mass Spectrometry for Classification of Amyloid Deposits. Amyloid.

[14] Leung N., Nasr S.H., Sethi S. (2012). How I Treat Amyloidosis: The Importance of Accurate Diagnosis and Amyloid Typing. Blood.

[15] Colombat M., Gaspard M., Camus M. (2022). Mass Spectrometry-Based Proteomics in Clinical Practice Amyloid Typing: State-of-the-Art from a French Nationwide Cohort. Haematologica.

[16] Sanchorawala V. (2024). Systemic Light Chain Amyloidosis. N. Engl. J..

[17] Turner R., Kalff A., Bergin K. (2022). The Utility of Euroflow MRD Assessment in Real-World Multiple Myeloma Practice. Front. Oncol..

[18] Flores-Montero J., van Dongen J.J.M., Orfao A. (2017). Next Generation Flow for Highly Sensitive and Standardized Detection of Minimal Residual Disease in Multiple Myeloma. Leukemia.

[19] Keren D.F., Bocsi G., Billman B.L. (2022). Laboratory Detection and Initial Diagnosis of Monoclonal Gammopathies. Arch. Pathol. Lab. Med..

[20] Palladini G., Russo P., Bosoni T. (2009). Identification of Amyloidogenic Light Chains Requires the Combination of Serum-Free Light Chain Assay with Immunofixation of Serum and Urine. Clin. Chem..

[21] Desjardins L., Liabeuf S., Lenglet A. (2013). Association between Free Light Chain Levels, and Disease Progression and Mortality in Chronic Kidney Disease. Toxins.

[22] Long T.E., Indridason O.S., Pálsson R. (2022). Defining New Reference Intervals for Serum Free Light Chains in Individuals with Chronic Kidney Disease: Results of the iStopMM Study. Blood Cancer J..

[23] Gudowska-Sawczuk M., Mroczko B. (2023). Free Light Chains κ and λ as New Biomarkers of Selected Diseases. Int. J. Mol. Sci..

[24] Paiva B., Vídriales M.B., Pérez J.J. (2011). The Clinical Utility and Prognostic Value of Multiparameter Flow Cytometry Immunophenotyping in Light-Chain Amyloidosis. Blood.

[25] Demyanets S., Kaider A., Simonitsch-Klupp I. (2020). Biological Properties of Bone Marrow Plasma Cells Influence Their Recovery in Aspirate Specimens: Impact on Classification of Plasma Cell Disorders and Potential Bias to Evaluation of Treatment Response. Ann. Hematol..

[26] Murugan R., Manivannan P., Gochhait D. (2024). Utility of Fine-Needle Aspiration Cytology Combined with Flow Cytometry in Extramedullary Hematolymphoid Lesions—A Cross-Sectional Study. CytoJournal.

[27] Li L., Sun Y., Luo J. (2024). Circulating Immune Cells and Risk of Osteosarcoma: A Mendelian Randomization Analysis. Front. Immunol..

[28] Lee T., Park C.J., Kim M. (2023). Evaluation of Laboratory Diagnostic Tests for Light-Chain Clonality and Bone Marrow Findings in AL Amyloidosis. Blood Res..

[29] Paiva B., Puig N., Lasa M. (2018). Flow Cytometry for Fast Screening and Automated Risk Assessment in Systemic Light-Chain Amyloidosis. Leukemia.

[30] Lisenko K., Schönland S.O., Jauch A. (2016). Flow Cytometry-Based Characterization of Underlying Clonal B and Plasma Cells in Patients with Light Chain Amyloidosis. Cancer Med..

[31] Kebel A., Röcken C. (2006). Immunohistochemical Classification of Amyloid in Surgical Pathology Revisited. Am. J. Surg. Pathol..

[32] Zhu Q., Sun J., An C. (2024). Mechanism of LncRNA Gm2044 in Germ Cell Development. Front. Cell Dev. Biol..

[33] Muchtar E., Jevremovic D., Dispenzieri A. (2017). The Prognostic Value of Multiparametric Flow Cytometry in AL Amyloidosis at Diagnosis and at the End of First-Line Treatment. Blood.

[34] Fotiou D., Dimopoulos M.A., Kastritis E. (2020). Systemic AL Amyloidosis: Current Approaches to Diagnosis and Management. HemaSphere.

[35] Azevedo E.P.C., Guimarães-Costa A.B., Torezani G.S. (2012). Amyloid Fibrils Trigger the Release of Neutrophil Extracellular Traps (NETs), Causing Fibril Fragmentation by NET-Associated Elastase. J. Biol. Chem..

[36] Himcinschi M.E., Uscatescu V., Gherghe G. (2024). The Role of Neutrophil Extracellular Traps in the Outcome of Malignant Epitheliomas: Significance of CA215 Involvement. Diagnostics.

[37] Kastritis E., Kostopoulos I.V., Theodorakakou F. (2021). Next Generation Flow Cytometry for MRD Detection in Patients with AL Amyloidosis. Amyloid.

[38] Chakraborty R., Hopson M., Divaya B. (2022). Impact of Bone Marrow Minimal Residual Disease Status on Quality of Organ Response in Systemic AL Amyloidosis. Am. J. Hematol..

[39] Staron A., Burks E.J., Lee J.C. (2020). Assessment of Minimal Residual Disease Using Multiparametric Flow Cytometry in Patients with AL Amyloidosis. Blood Adv..

[40] Li X., Huang B., Liu J. (2022). Clinical Value of Minimal Residual Disease Assessed by Multiparameter Flow Cytometry in Amyloid Light Chain Amyloidosis. J. Cancer Res. Clin. Oncol..

[41] Sidana S., Muchtar E., Sidiqi M.H. (2020). Impact of Minimal Residual Negativity Using Next Generation Flow Cytometry on Outcomes in Light Chain Amyloidosis. Am. J. Hematol..

[42] Muchtar E., Dispenzieri A., Jevremovici D. (2020). Survival Impact of Achieving Minimal Residual Negativity by Multi-Parametric Flow Cytometry in AL Amyloidosis. Amyloid.

[43] Paladini G., Paiva B., Orfao A. (2021). Minimal Residual Disease Negativity by Next-Generation Flow Cytometry Is Associated with Improved Organ Response in AL Amyloidosis. Blood Cancer J..

