# Supplementary material for: Proving Bone Marrow Plasma Cell Clonality by Flow Cytometry: An Important Tool in the Diagnosis of Immunoglobulin Light-Chain Amyloidosis
Source: Biomedicines. 2025 Nov 28;13(12):2925. doi: 10.3390/biomedicines13122925 (PMC12730388; doi:10.3390/biomedicines13122925)
Supplement: Supplementary file 1 [file biomedicines-13-02925-s001.zip › biomedicines-3898918-supplementary.pdf]

Supplementary Figures

Note: For all Figures in the supplementary material, the following statements are true: (i) outlier values represent incidental measurement variability and are not indicative of meaningful significance; (ii) Boxplots have the following: median, interquartile range, min, max and outliers; (iii) dFLC under 100 mg/L group consisted of 11 patients and dFLC over 100 mg/L group consisted of 12 patients.

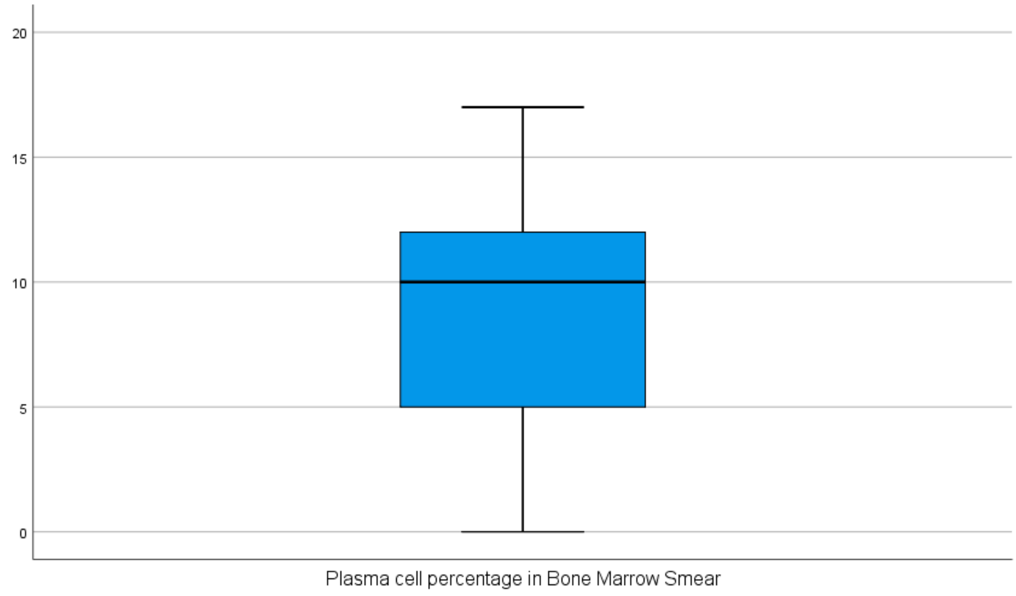

Figure S1. Plasma-cell percentage by bone marrow smear.
Boxplot of plasma-cell infiltration (%) from Wright–Giemsa–stained bone marrow smears in AL amyloidosis patients. Y-axis: Percentage of plasma cells from the total nucleated cells by microscopy. N=23.

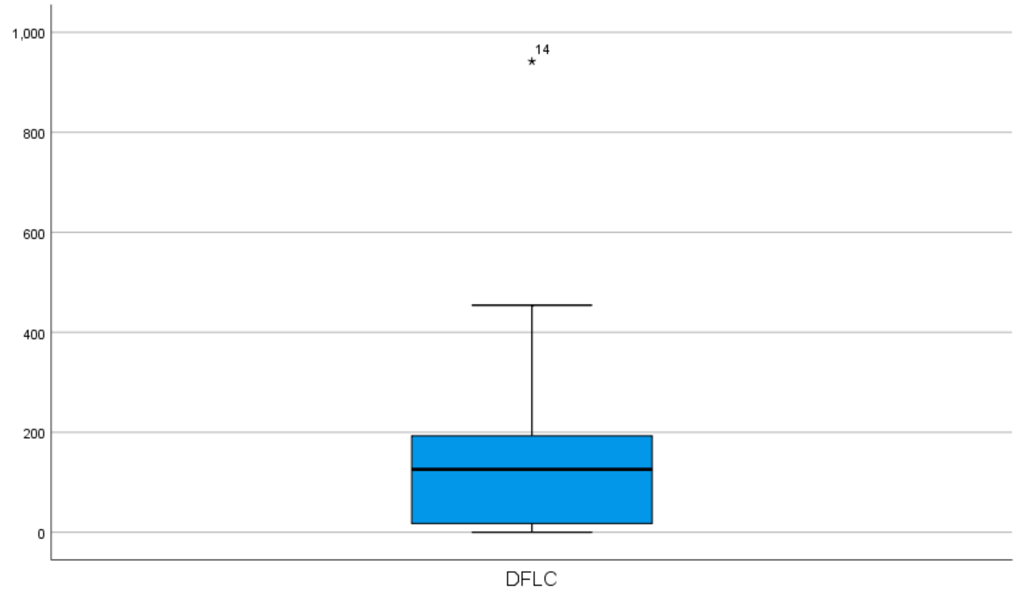

Figure S2. Distribution of dFLC values.
Boxplot of the difference between involved and uninvolved free light chains (dFLC, mg/L) in serum samples from n=23 patients with AL amyloidosis. Y-axis: value in mg/L of dFLC.

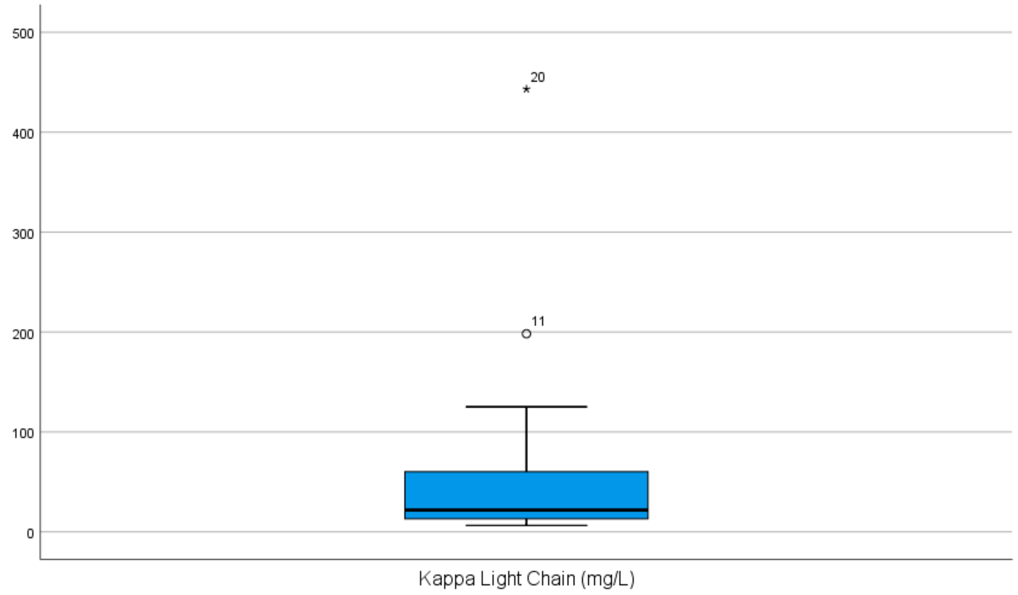

Figure S3. Serum κ light-chain concentration.
Boxplot of serum κ light-chain concentration (mg/L) in AL amyloidosis patients. Y-axis: Value in mg/L of Kappa Light chain in serum. N = 23.

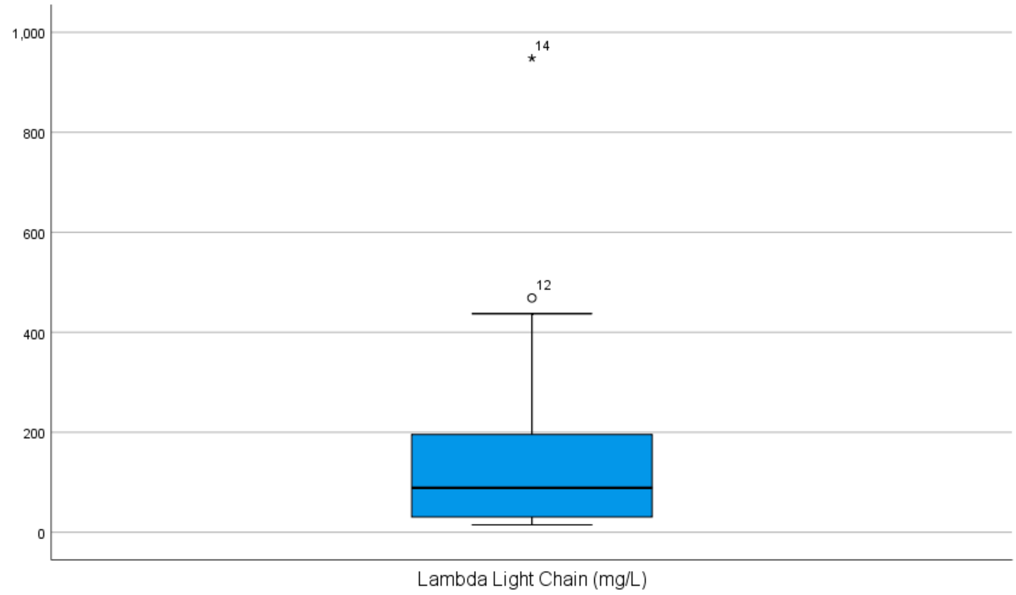

Figure S4. Serum λ light-chain concentration.
Boxplot of serum λ light-chain concentration (mg/L) in AL amyloidosis patients. Y-axis: Value in mg/L of Lambda Light chain in serum. N = 23.

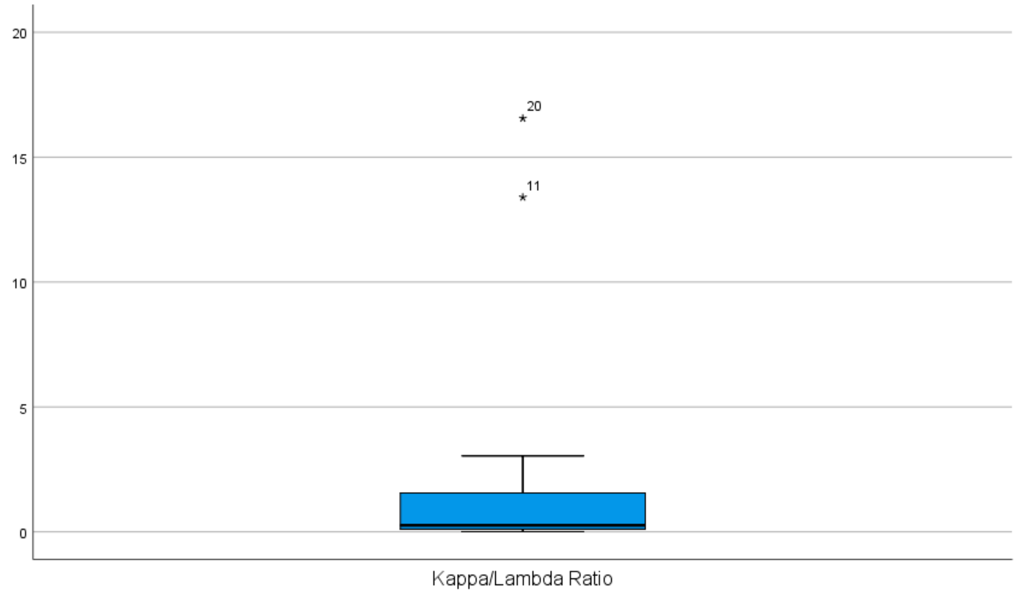

Figure S5. Serum κ/λ ratio.
Boxplot of serum κ/λ ratio in AL amyloidosis patients. Y-axis: the value of the Kappa/Lambda ratio. N = 23.

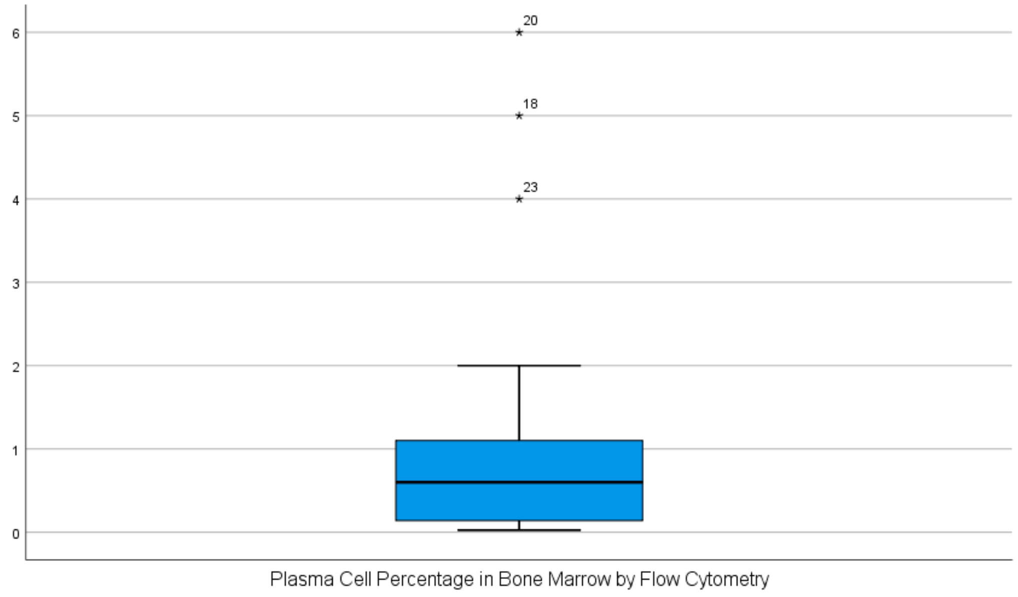

Figure S6. Plasma cell percentage in bone marrow by flow cytometry.
Boxplot of plasma cell infiltration (%) measured in bone marrow aspirates using multiparameter flow cytometry. Y-axis: Percentage of Plasma Cells from total white blood cells and precursors by flow cytometry. N = 23.

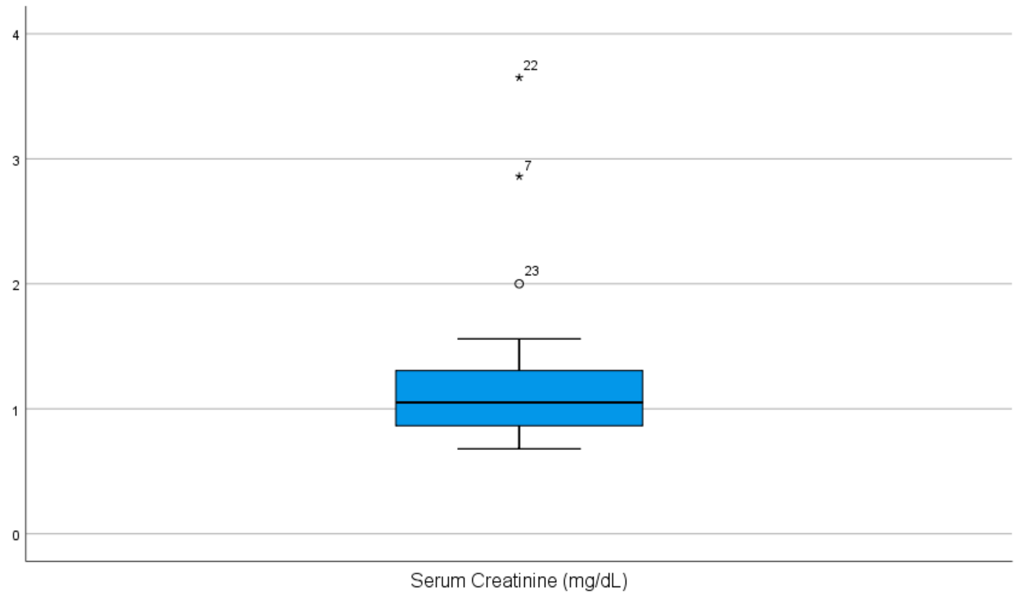

Figure S7. Serum creatinine in AL amyloidosis patients.
Boxplot of serum creatinine values (mg/dL) in the cohort. Y-axis: Value in mg/dL of the serum creatinine. N = 23.

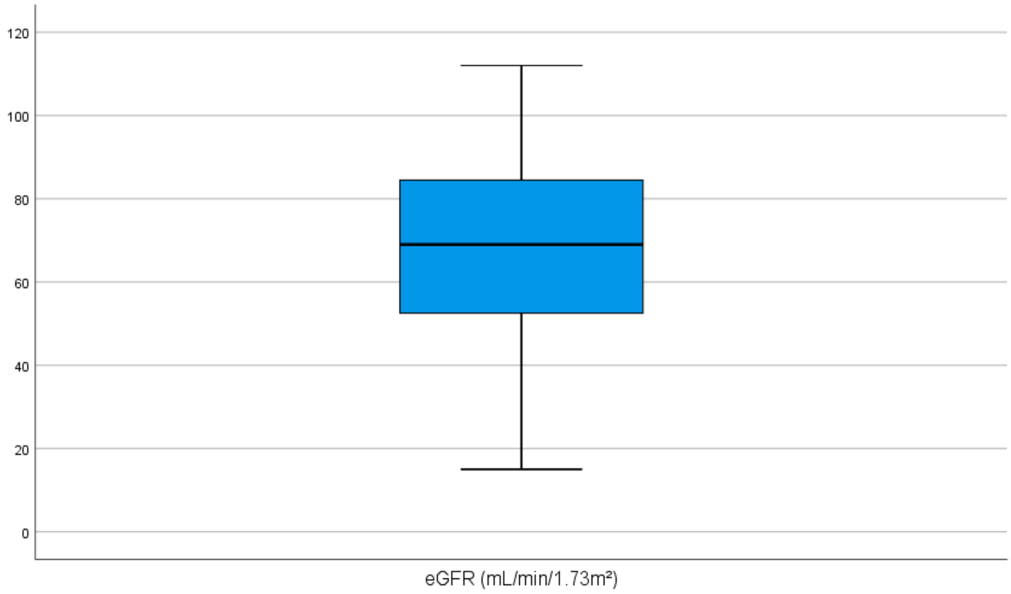

Figure S8. Estimated glomerular filtration rate (eGFR) in AL amyloidosis patients.
Boxplot of eGFR (mL/min/1.73 m²) derived from serum creatinine in N = 23 patients. Values calculated using CKD-EPI formula. Y-axis: Value of eGFR in ml/min/1.73 m²).

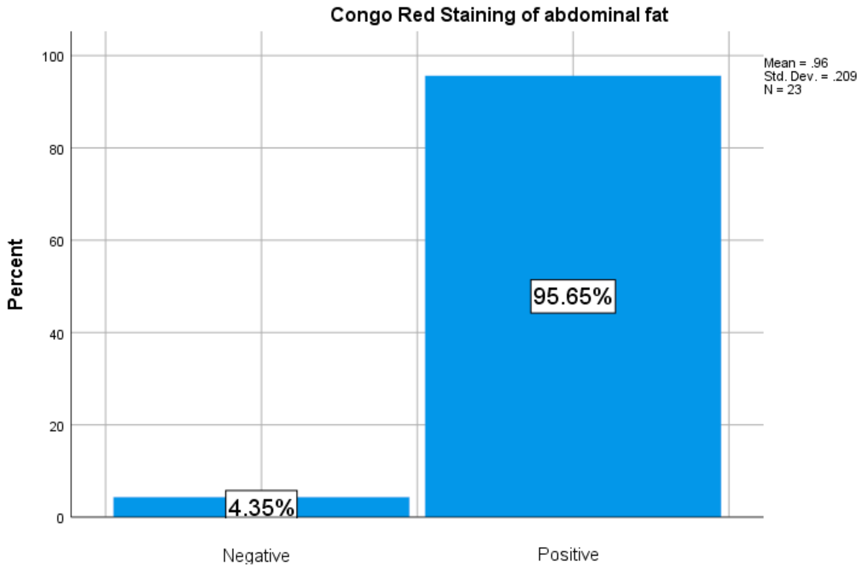

Figure S9. Congo red staining of abdominal fat and salivary gland.
Bar chart showing the proportion of positive and negative Congo red staining in abdominal fat biopsies. Staining confirmed amyloid deposition in 95.65% of cases (n=22). N=1 patient (4.35%) was negative and later confirmed by electronic microscopy.

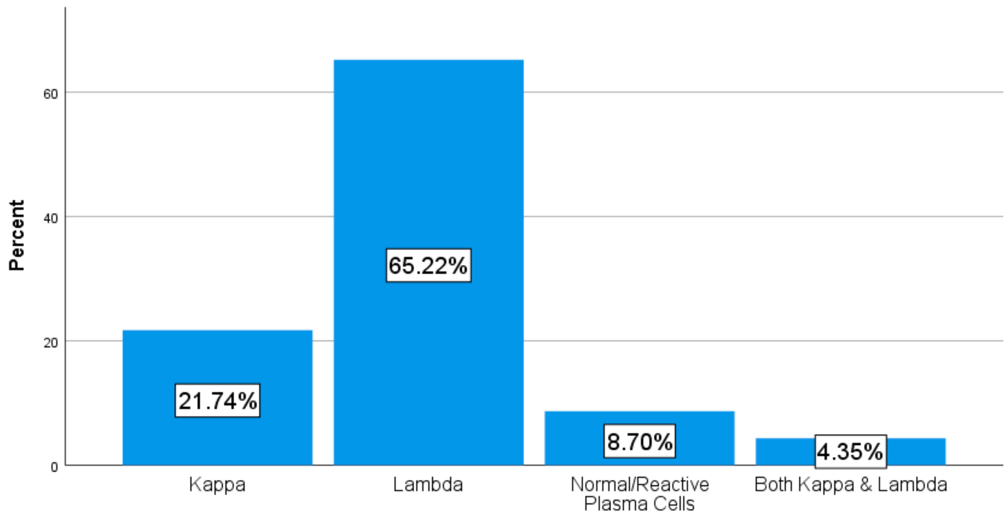

Figure S10. Distribution of κ and λ light-chain clonality detected by flow cytometry.
Bar chart showing proportions of clonal κ, clonal λ, dual κ+λ, and normal/reactive plasma cells in bone marrow aspirates analyzed by multiparameter flow cytometry. λ restriction predominated (65.2%). N = 23. κ was found in 21.74% (5 patients), λ was found in 65.22% (15 patients), 4.35% (1 patient) had 2 clones, 1 λ and 1 κ, and 8.7% (2 patients) were normal/reactive by flow cytometry.

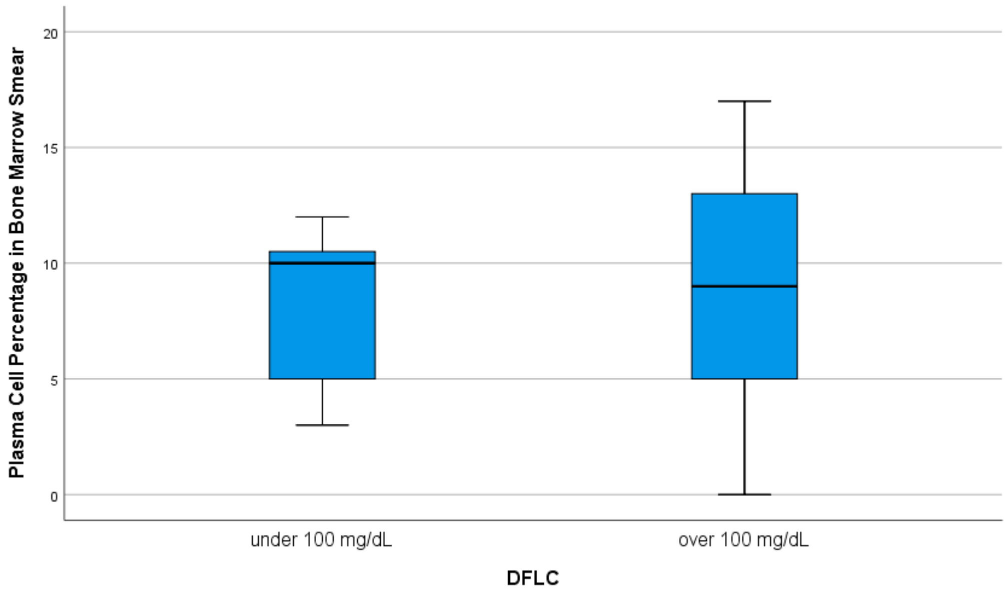

Figure S11. Plasma-cell percentage by bone marrow smear in dFLC subgroups.
Boxplot of plasma cell infiltration (%) from Wright–Giemsa–stained bone marrow smears in patients with dFLC < 100 mg/L and ≥ 100 mg/L.

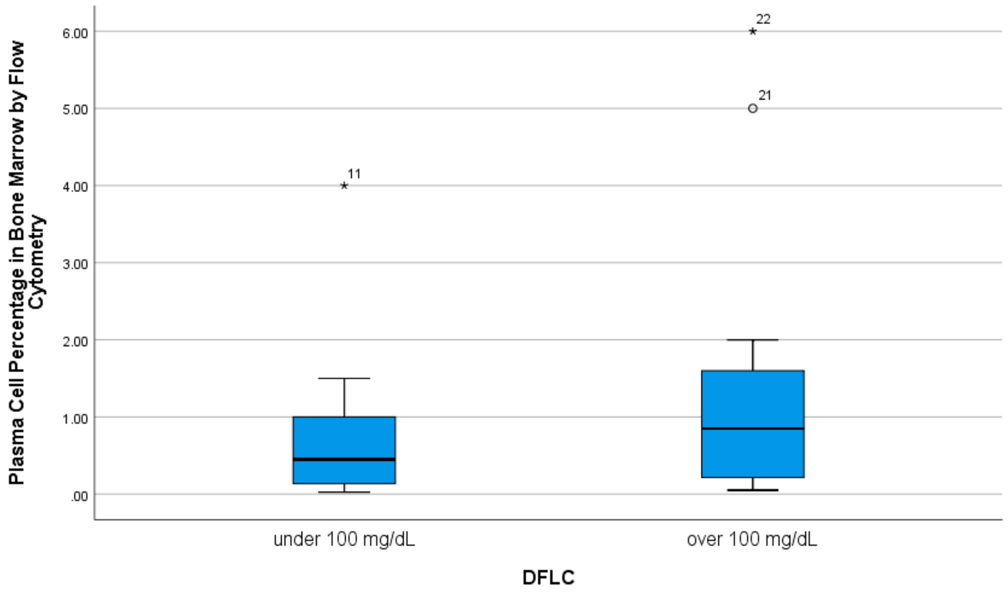

Figure S12. Plasma cell percentage by flow cytometry in dFLC subgroups.
Boxplot of bone marrow plasma cell infiltration (%) determined by multiparameter flow cytometry in patients with dFLC < 100 mg/L and ≥ 100 mg/L.

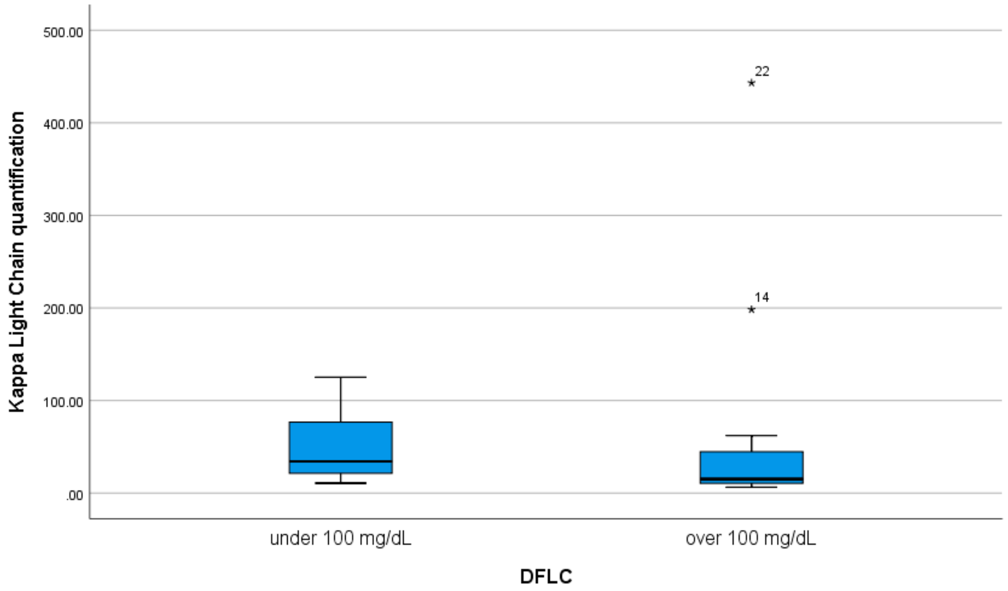

Figure S13. Serum κ light chain quantification in dFLC subgroups.
Boxplot showing serum κ light chain concentration (mg/L) in patients with dFLC < 100 mg/L and ≥ 100 mg/L.

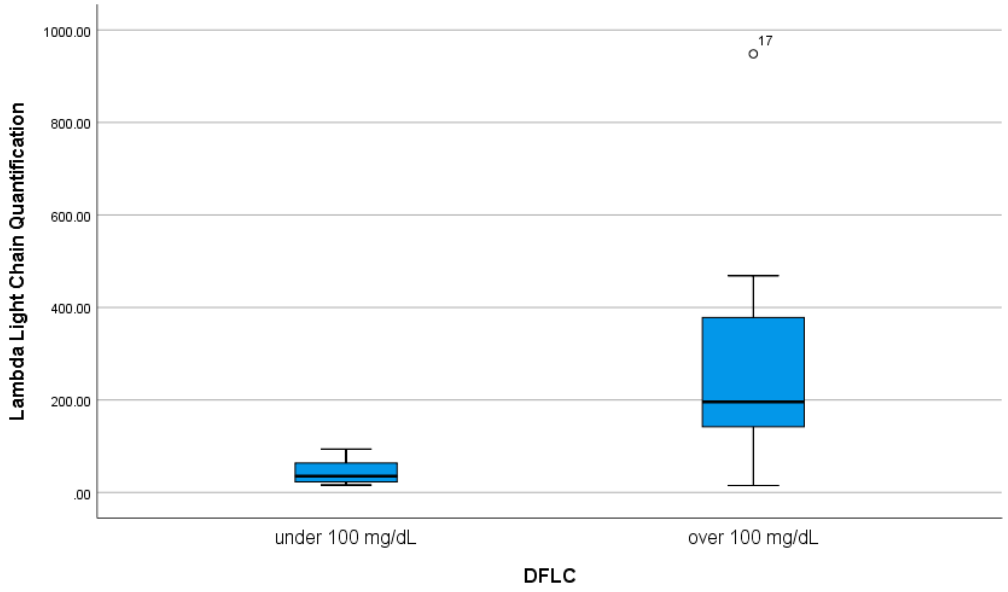

Figure S14. Serum λ light chain quantification in dFLC subgroups.
Boxplot of serum λ light chain concentration (mg/L) in patients with dFLC < 100 mg/L and ≥ 100 mg/L.

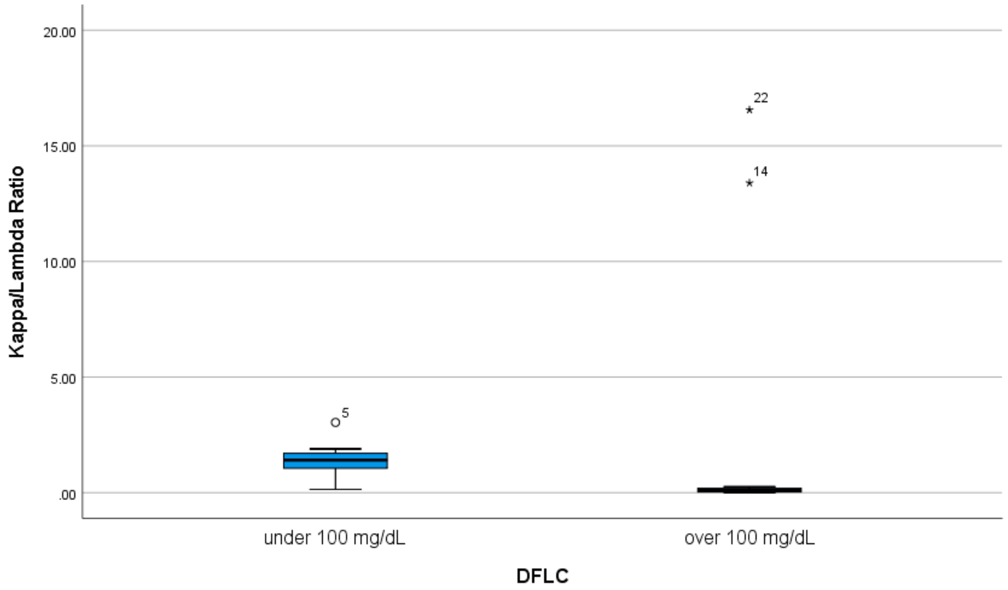

Figure S15. Serum κ/λ ratio in dFLC subgroups.
Boxplot of serum κ/λ ratio in patients with dFLC < 100 mg/L and ≥ 100 mg/L.

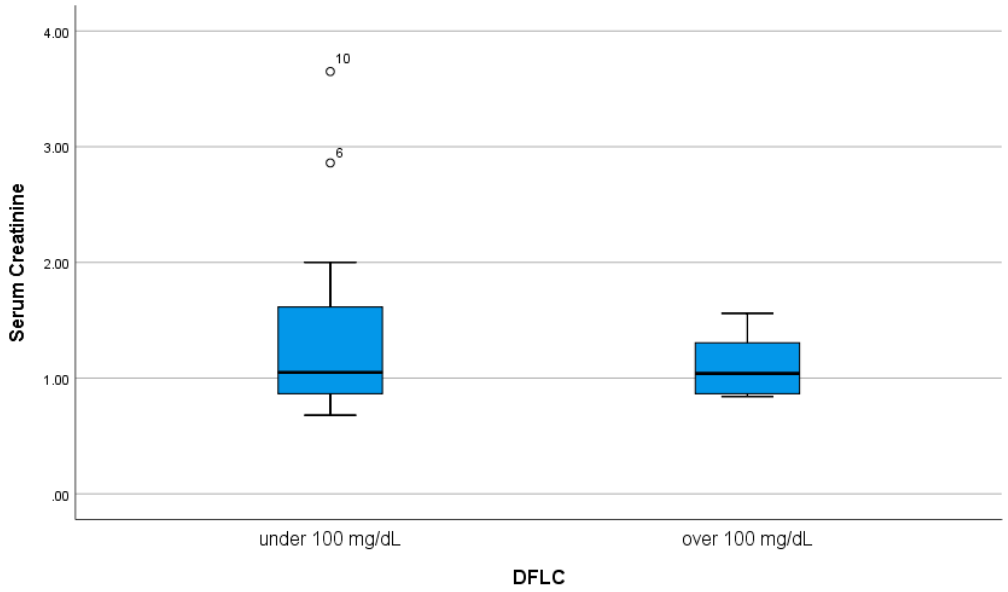

Figure S16. Serum creatinine in dFLC subgroups.
Boxplot of serum creatinine (mg/dL) in patients with dFLC < 100 mg/L and ≥ 100 mg/L.
